# Design and Characterisation of Inhibitory Peptides against Bleg1_2478, an Evolutionary Divergent B3 Metallo-β-lactamase

**DOI:** 10.3390/molecules25245797

**Published:** 2020-12-09

**Authors:** Gayathri Selvaraju, Thean Chor Leow, Abu Bakar Salleh, Yahaya M. Normi

**Affiliations:** 1 Enzyme and Microbial Technology Research Center, Faculty of Biotechnology and Biomolecular Sciences, Universiti Putra Malaysia, Serdang 43400, Malaysia; gs43977@student.upm.edu.my (G.S.); adamleow@upm.edu.my (T.C.L.); abubakar@upm.edu.my (A.B.S.); 2 Department of Cell and Molecular Biology, Faculty of Biotechnology and Biomolecular Sciences, Universiti Putra Malaysia, Serdang 43400, Malaysia

**Keywords:** inhibitory peptide, Bleg1_2478, B3 subclass metallo-β-lactamase, docking, inhibition, active site

## Abstract

Previously, a hypothetical protein (HP) termed Bleg1_2437 (currently named Bleg1_2478) from *Bacillus lehensis* G1 was discovered to be an evolutionary divergent B3 subclass metallo-β-lactamase (MBL). Due to the scarcity of clinical inhibitors for B3 MBLs and the divergent nature of Bleg1_2478, this study aimed to design and characterise peptides as inhibitors against Bleg1_2478. Through in silico docking, RSWPWH and SSWWDR peptides with comparable binding energy to ampicillin were obtained. In vitro assay results showed RSWPWH and SSWWDR inhibited the activity of Bleg1_2478 by 50% at concentrations as low as 0.90 µM and 0.50 µM, respectively. At 10 µM of RSWPWH and 20 µM of SSWWDR, the activity of Bleg1_2478 was almost completely inhibited. Isothermal titration calorimetry (ITC) analyses showed slightly improved binding properties of the peptides compared to ampicillin. Docked peptide–protein complexes revealed that RSWPWH bound near the vicinity of the Bleg1_2478 active site while SSWWDR bound at the center of the active site itself. We postulate that the peptides caused the inhibition of Bleg1_2478 by reducing or blocking the accessibility of its active site from ampicillin, thus hampering its catalytic function.

## 1. Introduction

β-lactam antibiotics have been used widely as frontline therapeutics in treating bacteria-related infections and diseases. These molecules specifically target bacterial pathogens by interfering with bacterial cell wall synthesis, which will eventually cause cell lysis [1]. However, the emergence of antimicrobial resistance (AMR) among bacterial pathogens had raised major concerns in global public health as it can render the commonly used antibiotics and antimicrobial therapy ineffective, prolonging hospital stay and increasing medical expenses. Severe cases of AMR can lead to more complicated medical procedures such as surgery to remove the focal point of infection and even untimely deaths. As such, AMR will exert a huge impact on the world economy in the future if the current situation is not tackled [2].

AMR among bacteria can be acquired through various means, i.e., through: (1) hydrolysis or inactivation of antibiotics by synthesizing enzymes [3]; (2) redox process by exploiting the oxidation or reduction of the antibiotics [4]; (3) modification of antibiotics by chemical substitution [5]; (4) modification of the inhibition binding site in the target [6]; (5) mutations on genes that encode the target or efflux pump that affect antibiotics uptake [7,8]; (6) horizontal gene transfer where resistant genes are transferred from one pathogen to another via transduction, conjugation or transformation [9]. Mechanism (1) is one of the most well-studied AMR mechanisms which involves β-lactamase enzymes. β-lactamases deactivates β-lactam antibiotics by hydrolysing the β-lactam ring of the β-lactam antibiotics. Based on the Ambler classification scheme, β-lactamases are classified as Class A, B, C, and D based on their sequence similarities. Class A, C and D β-lactamase are categorised as serine-β-lactamase (SBLs) while Class B β-lactamases are categorised as metallo-β-lactamases (MBLs) [10]. MBLs can be further divided into four subclasses, B1, B2, B3 and B4, based on their sequence homology [11]. All MBLs are dependent on Zn^2+^ metal ions as co-factor for catalysis. B1, B3 and B4 MBLs require two Zn^2+^ ions for their catalytic activity, while B2 MBLs require only one Zn^2+^ ion for their activity [12,13,14,15,16]. Compared to SBLs, MBLs pose a greater threat to public health due to their broad substrate specificity. Clinically available inhibitors such as clavulanate, sulbactam and tazobactam are effective against Class A SBLs [17,18,19], but not MBLs [20]. Hence, they have garnered attention in the recent decade due to their inability to be inhibited by commonly used clinical inhibitors or drug combinations [21].

Previously, a hypothetical protein termed Bleg1_2437 (currently renamed as Bleg1_2478), which has a comparable sequence identity to MBL in the range of 43–65%, was discovered from the pool of hypothetical proteins of *Bacillus lehensis* G1 alkaliphile. Its predicted in silico structure revealed similarity to the αββα fold and global topology of MBLs. Analysis of its active site and metal-binding ligands revealed that similarity to B3 MBL. Biochemical characterization of purified recombinant Bleg1_2478 further confirmed that it was indeed capable of degrading β-lactam antibiotics, with ampicillin as the preferred substrate [22]. However, in terms of evolutionary relationship, it did not exhibit relatedness to other B3 MBLs [22]. In view of limited inhibitors against B3 MBLs to date and the evolutionary divergent nature of Bleg1_2478, this study aims to design inhibitory peptides against Bleg1_2478 B3 subclass MBL and characterise their inhibitory potential and properties through in vitro and in silico approaches. This is one of few reports on peptide inhibitors targeted against B3 MBLs [23,24].

## 2. Results

### 2.1. Fixed and Random Docking of Ampicillin against Bleg1_2478

**Figure 1:**
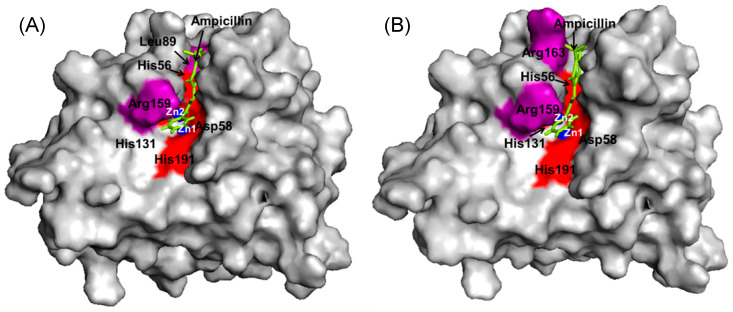
(**A**) Fixed docking of ampicillin and Bleg1_2478 with binding energy of 8.52 kcal/ mol. (**B**) Random docking of ampicillin and Bleg1_2478 with binding energy of 8.15 kcal/ mol. Both docking analysis via YASARA showed interaction at its active site which plays an important role in ampicillin degradation. (The metal-binding residues are shown in red colour; non-active site residues are shown in purple colour; blue spheres represent Zn^2+^; green stick represents ligand (ampicillin).

Through both fixed and random docking analysis of ampicillin onto previously built dizinc Bleg1_2478 protein model [22] using YASARA [25], results showed that ampicillin bound to Bleg1_2478 with a comparable binding energy of 8.52 and 8.15 kcal/mol respectively. Docking results specifically suggested that there was only one specific binding site in Bleg1_2478 for ampicillin, which was at the active site which houses the metal-binding residues important for its catalytic activity (Figure 1). Both bindings involved metal-binding residues of Bleg1_2478 which are His54, His56, Asp58, His59, His131 and His191, and other residues namely Leu89, Arg159 and Arg163, as well as Zn1; through hydrophobic, cation–π, π–π and hydrogen bond interactions (Table 1 and Table 2).

### 2.2. Design and Docking of Inhibitory Peptides against Bleg1_2478

Several peptides were retrieved from the Collection of Anti-Microbial Peptides (CAMPR3) online database [26] and were firstly docked against Bleg1_2478 via random docking using YASARA. Peptides RRAARF, RRWFWR and RRWWFR, which showed a comparable range of binding energy with that of ampicillin (Table 3), were chosen to be derivatised. This was achieved by substituting certain amino acids in the peptides to be subsequently docked onto Bleg1_2478 for analysis of their binding energies.

As a result, RYWPRF and RYTPRF were derived from RRAARF, while RSWNWH, RSWCWH and RSWPWH were derived from RRWFWR, and SRWWDR, SRWWYR and SSWWDR were derived from RRWWFR. Most of these peptides exhibited better binding energy and affinity to ampicillin when docked to Bleg1_2478 via both fixed and random docking (Table 4). Comparing fixed docking results generated by YASARA [25] and AutoDock Vina [27], they corroborated well with one another, with the exception of peptides RSWNWH, RRWWSR, SRWWDR, SRWWYR and SSWWDR. Fixed docking results from YASARA for RRWWSR showed that it did not bind at Bleg1_2478 active site but at a secondary site far from the active site, identified through random docking. This accounted for the difference in the binding energies observed for this particular peptide (Table 4). Based on these observations, a clear conclusion could not be drawn to specifically pinpoint which peptides would exhibit the best inhibitory properties towards Bleg1_2478 compared to ampicillin. Hence, all peptides were subjected to synthesis and assay.

### 2.3. Overexpression and Purification of Bleg1_2478 Recombinant Protein

**Figure 2:**
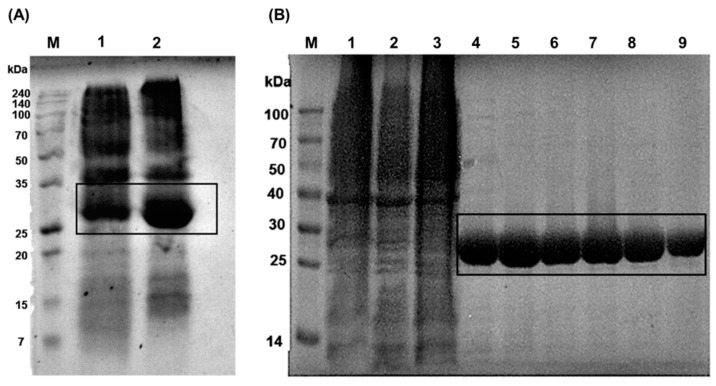
(**A**) Bleg1_2478 recombinant protein expression in *E. coli* BL21 (DE3). M: Protein Marker; Lane 2: pellet and Lane 3: supernatant fractions. The protein size of the recombinant Bleg1_2478 protein is 26 kDa. (**B**) Purification of Bleg1_2478 via affinity chromatography. M: Protein Marker; Lane 1–Lane 3: Flow-through; Lane 4–Lane 9: purified fraction of Bleg1_2478 recombinant protein.

Prior to the inhibitory assay of the designed peptides, Bleg1_2478 recombinant protein was expressed in *Escherichia coli* BL21(DE3) in LB media containing 0.1 mM isopropyl-β-d-thiogalactopyranoside (IPTG) and 100 µM ZnSO_4_ after cultivation for 18 h at 20 °C. Most of the recombinant protein (corresponding to protein size of 26 kDa in the sodium dodecyl sulphate polyacrylamide electrophoresis (SDS PAGE) gel) was expressed in the soluble fraction (Figure 2A), with 2.1 mg/mL of protein obtained. Purification of the soluble fraction yielded a distinct 26 kDa protein band (Figure 2B). Dialysis and concentration of the purified recombinant Bleg1_2478 protein yielded a protein concentration of 27 mg and was subsequently used for inhibitory assay with the designed peptides.

### 2.4. Inhibitory Assay of Designed Peptides against Bleg1_2478

**Figure 3:**
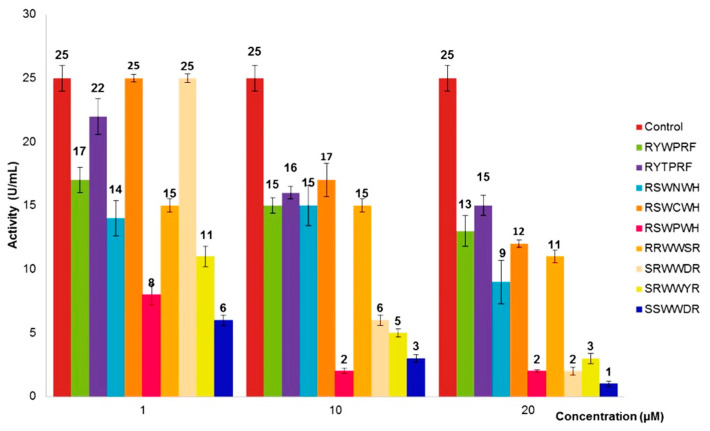
Inhibitory effect of designed peptides on the activity of Bleg1_2478.

The inhibitory activities of the peptides towards Bleg1_2478 were tested at concentrations of 1, 10 and 20 µM respectively. Without the addition of peptides, the activity of Bleg1_2478 (control) was recorded to be 25 U/mL (Figure 3). With the addition of 1 µM of peptides, peptides RSWPWH and SSWWDR showed more than 50% inhibitory activity towards Bleg1_2478. At this concentration, peptides RSWPWH and SSWWDR reduced Bleg1_2478 activity to 8 U/mL (68% inhibition) and 6 U/mL (76% inhibition), respectively (Figure 3). A further increase in the concentration of these peptides to 10 µM resulted in further inhibition of Bleg1_2478 activity to 2 U/mL (92% inhibition) and 3 U/mL (88% inhibition), respectively. At 20 µM, SSWWDR achieved its highest inhibition of Bleg1_2478 by 96% while the effect of RSWPWH remained the same (Figure 3). Hence, based on the results obtained, it can be concluded that SSWWDR noticeably inhibited Bleg1_2478 activity by 76% inhibition even at a concentration as low as 1 µM. It reached its highest inhibitory potential at 20 µM, whereby Bleg1_2478 enzymatic activity was almost nullified. RSWPWH, on the other hand, exerted its best inhibitory potential at 10 µM with no noticeable changes above this concentration.

**Figure 4:**
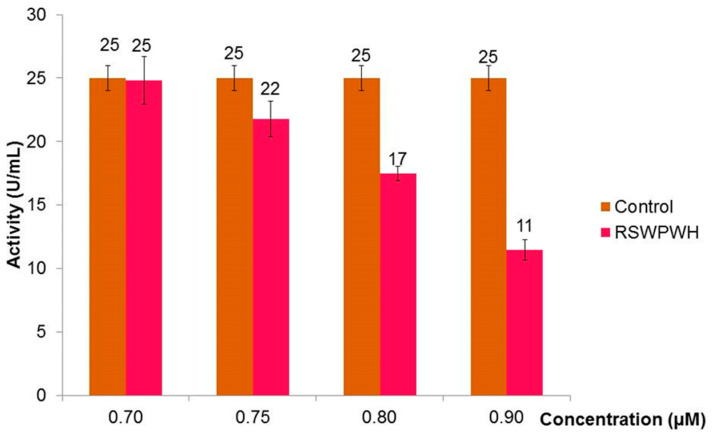
Inhibitory effect of designed peptide RSWPWH on the activity of Bleg1_2478 at 0.70, 0.75, 0.80 and 0.90 µM respectively.

**Figure 5:**
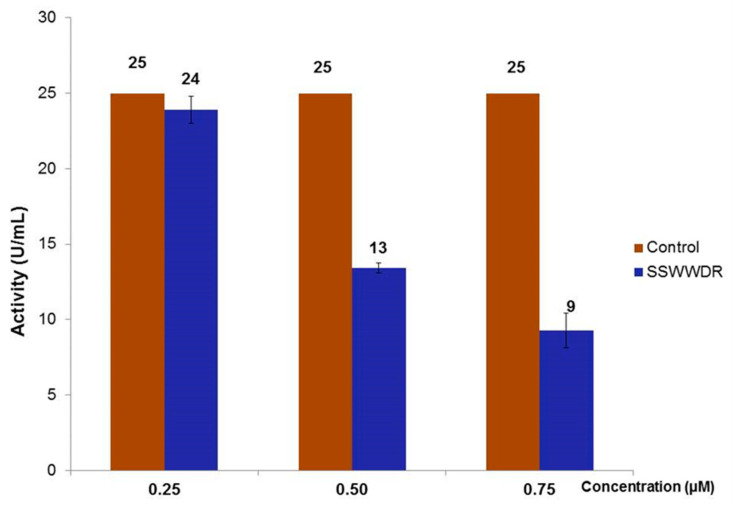
Inhibitory effect of designed peptide SSWWDR on the activity of Bleg1_2478 at concentrations of 0.25, 0.50 and 0.75 µM respectively.

As RSWPWH and SSWWDR exhibited significant inhibition than other designed peptides, they were chosen to be tested at concentrations lower than 1 µM. In the case of RSWPWH, the peptide exhibited slight inhibition of Bleg1_2478 by 12% at 0.75 µM (Figure 4). It exerted 54% of inhibition at 0.90 µM (Figure 4). Interestingly, for SSWWDR, significant inhibition of Bleg1_2478 by 64% was observed at 0.75 µM (Figure 5), indicating higher potency of the peptide compared to RSWPWH. Hence, SSWWDR was further tested at 0.25 and 0.50 µM, respectively to identify the least concentration needed for the peptide to exert minimal inhibition on Bleg1_2478. Results showed that the peptide was able to inhibit Bleg1_2478 as low as 4% at 0.25 µM. The inhibition increased by more than 10-fold, i.e., by 48% when the concentration of the peptide was doubled to 0.50 µM (Figure 5). As such the minimum inhibitory concentration to cause 50% inhibition of Bleg1_2478 (IC_50_) is 0.90 µM for RSWPWH and 0.50 µM for SSWWDR (Table 5). As results from the assay proved that both RSWPWH and SSWWDR peptides were able to inhibit Bleg1_2478 even at a concentration lower than 1 µM, they were hence chosen for further detailed analysis.

### 2.5. Isothermal Titration Calorimetry (ITC) Analysis of Peptides with Bleg1_2478

ITC was carried out for protein–ampicillin and protein–peptide mixtures to determine their binding properties. Based on the results obtained, both RSWPWH and SSWWDR inhibitory peptides showed better binding affinity (K_a_) and strength (K_d_) towards Bleg1_2478 compared to ampicillin (Table 6). RSWPWH inhibitory peptide showed a three-fold increase in its binding affinity towards Bleg1_2478 while SSWWDR recorded a 1.5-fold increase in its binding affinity towards the protein. More significant is the binding strength of these peptides whereby they exhibited a respective 34 to 68-fold increase in this aspect compared to ampicillin. Based on the stoichiometric values (n) obtained, both of the inhibitory peptides bind to Bleg1_2478 at one binding site, similar to ampicillin.

**Figure 6:**
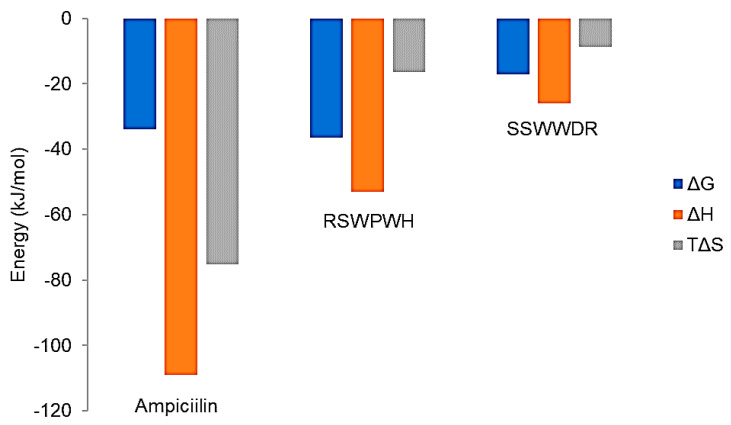
The binding signature (free energy, binding enthalpy, and entropy factor) plotted for the binding events involving ampicillin and the inhibitory peptides.

Binding signature plot (Figure 6) showed that the binding of the inhibitory peptides to Bleg1_2478 comprised hydrogen bonding and hydrophobic interactions as indicated by the negative or favourable binding enthalpy (ΔH) and entropy factor (TΔS). The binding of both inhibitory peptides with Bleg1_2478 was exothermic in nature, as denoted by their negative Gibbs free energy values. The binding involving peptide RSWPWH showed a comparable free energy value with ampicillin while the binding involving peptide SSWWDR recorded a decrease in the free energy value, signifying that the interaction was less spontaneous than those involving ampicillin and peptide RSWPWH.

### 2.6. Docking Analysis of Peptide-Bleg1_2478 Interaction

Random and fixed docking of inhibitory peptide RSWPWH to Bleg1_2478 gave forth comparable binding energy of 8.34 and 8.93 kcal/mol, respectively. Inspection of the residues from the peptide that interacted with Bleg1_2478 showed that only Arg-1, Trp-3 and Trp-5 were involved; forming interactions that include hydrophobic, π–π, cation–π, H-bond interactions (Table 7). Inspection of the residues from Bleg1_2478 involved in the interaction showed that none of the residues are those of the metal-binding ligands of Bleg1_2478.

**Figure 7:**
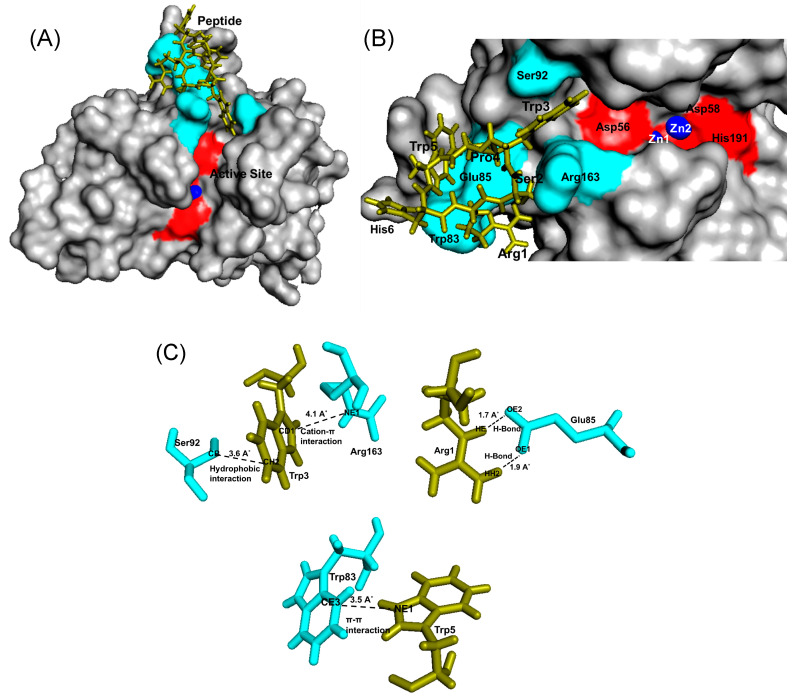
(**A**) Bleg1_2478 docked with RSWPWH peptide via random docking using YASARA. (**B**) The binding site of RSWPWH on Bleg1_2478. (**C**) Interaction between residues of Bleg1_2478 and RSWPWH peptide during global random docking. Trp3 forms a hydrophobic bond with Ser92 and cation–π interaction with Arg163; Arg1 forms two hydrogen bonds with Glu85; Trp5 forms π–π interaction with Trp83. The active site residues of Bleg1_2478 are highlighted in red; the non-active site residues are highlighted in turquoise. Zn^2+^ ions are represented by blue spheres. Peptide is represented as green sticks.

Analysis on the possible binding site on the protein predicted through random docking revealed that peptide RSWPWH binds to Bleg1_2478 at a site slightly away from the active site (Figure 7A,B) and involved residues within that vicinity, mainly Trp83, Glu85, Ser92 and Arg163 (Figure 7B,C).

**Figure 8:**
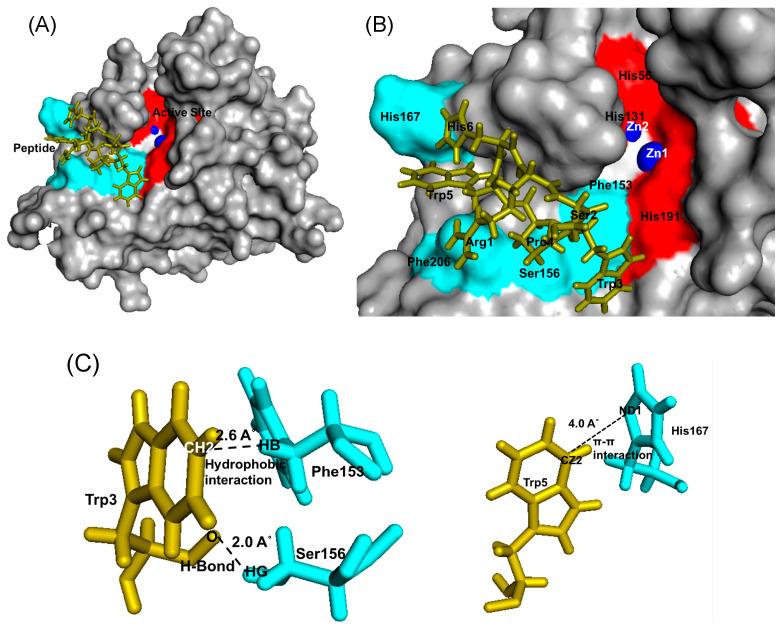
(**A**) Bleg1_2478 docked with RSWPWH peptide via fixed docking using YASARA. (**B**) The binding site of RSWPWH on Bleg1_2478. (**C**) Interaction between residues of Bleg1_2478 and RSWPWH during fixed docking. Trp3 forms hydrogen bond with Ser156 and hydrophobic bond with Phe153. Trp5 forms π–π bond with His167 and Arg1 forms cation–π interaction with Phe206. Active site residues of Bleg1_2478 are highlighted in red; non-active site residues are highlighted in turquoise. Zn^2+^ ions are represented by blue spheres. Peptide is represented by green stick.

Fixed docking analysis on the other hand showed that the peptide bound to Bleg1_2478 within the vicinity of the active site, without involving the catalytic residues His54, His56, Asp58, His59, His131 and His191 (Figure 8A,B). The results showed that three residues from the RSWPWH peptide (i.e., Arg1, Trp3 and Trp5) specifically interacted with Phe153, Ser156, His167 and Phe206 residues (Figure 8C, Table 6). Trp3 interacted with both Ser156 and Phe153 by forming hydrogen and hydrophobic bonds, respectively. Trp5 forms π–π bond with His167 and Arg1 formed cation–π interaction with Phe206.

**Figure 9:**
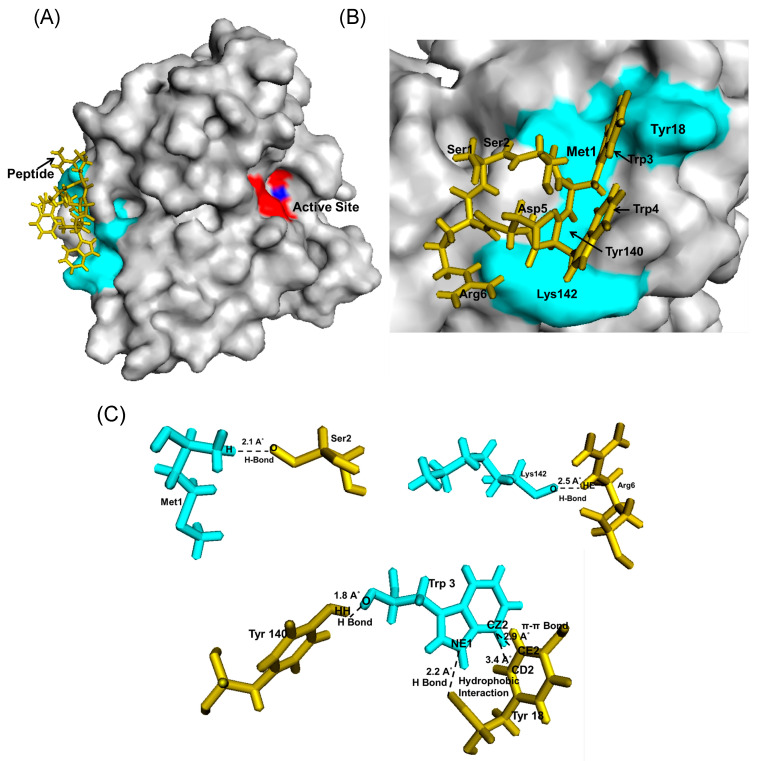
(**A**) Bleg1_2478 docked with SSWWDR via random docking using YASARA. (**B**) The binding site of SSWWDR on Bleg1_2478. (**C**) Interaction between residues of Bleg1_2478 and SSWWDR during random docking. Trp3 forms hydrogen, π–π and hydrophobic bonds with Tyr18 and another hydrogen bond with Tyr140; Ser2 forms hydrogen bond with Met1; and Arg6 forms a hydrogen bond with Lys142. Active site residues of Bleg1_2478 are highlighted in red; non-active site residues are highlighted in turquoise. Zn^2+^ ions are represented by blue spheres. Peptide is represented by green sticks.

For Bleg1_2478 interaction involving peptide SSWWDR, both random and fixed docking analysis gave forth binding energy of 6.15 and 6.47 kcal/mol, respectively. Further analysis on the residues from the peptide and protein involved in the interaction showed that none of the residues from both molecules were similarly involved in both dockings (Table 8). Analysis on Bleg1_2478-SSWWDR complex generated via random docking revealed a possible peptide binding site that was very far from Bleg1_2478 active site (Figure 9A,B), involving amino acids at the N-terminal of the protein such as Met1 and Tyr18, in addition to Tyr140 and Lys142 (Figure 9C, Table 8). Three amino acids from the peptide, i.e., Ser2, Trp3 and Arg6 were involved in the interaction with Bleg1_2478. Trp3 exhibited the most important interaction from its side chain atoms with Bleg1_2478 via Tyr18 and Tyr140 (Figure 9C, Table 8).

**Figure 10:**
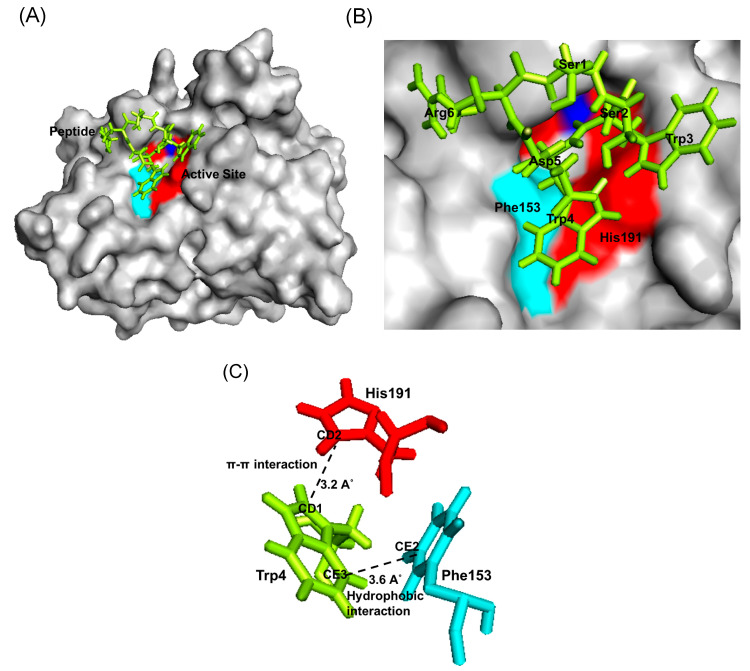
(**A**) Bleg1_2478 docked with SSWWDR via fixed docking using YASARA. (**B**) The binding site of SSWWDR on Bleg1_2478. (**C**) Interaction between residues of Bleg1_2478 and SSWWDR during docking via fixed docking. Trp4 forms a hydrophobic bond with Phe153 and π–π interaction with His191. Active site residues of Bleg1_2478 are highlighted in red; non-active site residues are highlighted in turquoise. Zn^2+^ ions are represented by blue spheres. Peptide is represented by green sticks.

However, analysis on the Bleg1_2478-SSWWDR complex generated via fixed docking revealed that the peptide bound to the center of the active site of the protein (Figure 10A,B); whereby only Trp4 from the peptide interacted with Phe153 and His191 forming hydrophobic and π–π interactions respectively (Figure 10C, Table 8).

### 2.7. Prediction of Physicochemical Properties of Inhibitory Peptides

Several physical properties of the inhibitory peptides were computed. Both inhibitory peptides shared comparable characteristics in terms of their diameter, approximate volume and total hydrophobic ratio (Table 9). The significant differences between the peptides are their molecular weight, net charge at pH 7.0, solubility and stability. RSWPWH has a net charge of +1.1 at pH 7.0 while peptide SSWWDR has a zero net charge at the same pH. SSWWDR has better solubility than RSWPWH due to a higher number of hydrophilic residues. In addition, SSWWDR also has better stability (Table 9).

## 3. Discussion

**Figure A1:**
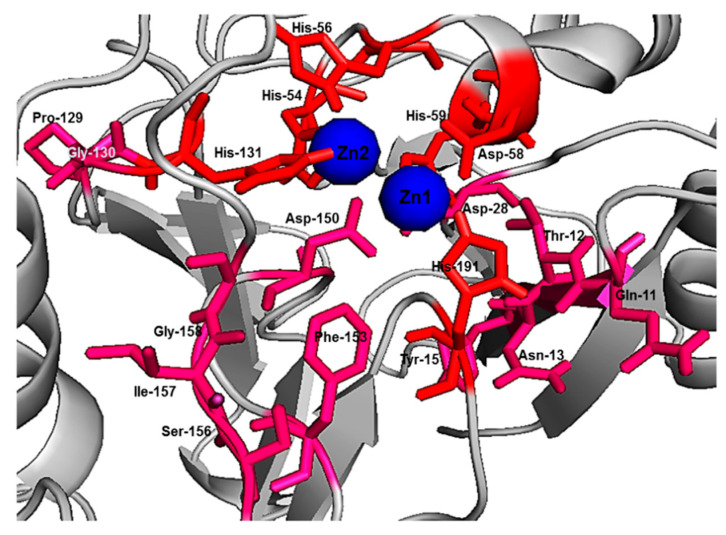
Close-up view of the active site of Bleg1_2478 consisting of metal-binding ligands (His-54, His-56, Asp-58, His-59, His-131 and His-191) and other amino acids (Gln-11, Thr-12, Asn-13, Tyr-15, Asp-28, Pro-129, Gly-130, Asp-150, Phe-153, Ser-156, Ile-157, and Gly-158).

**Figure A2:**
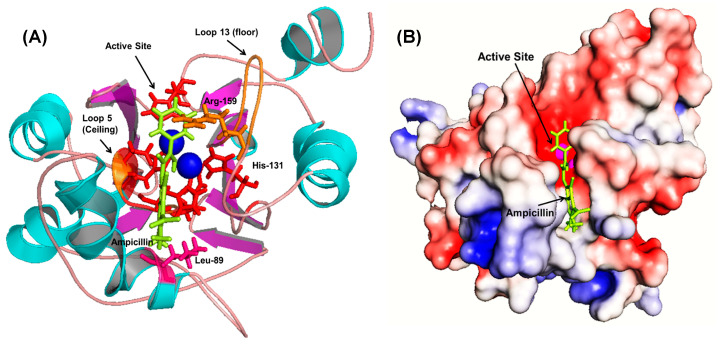
Interaction of ampicillin with important structural features of Bleg1_2478 in (**A**) ribbon and (**B**) hydrophobic surface representations.

Initial fixed and random docking analyses of ampicillin with Bleg1_2478 were undertaken to determine the binding properties of the β-lactam antibiotic to the protein. Within a distance of 5.0 Å from the binding site, results from both docking analysis showed whilst Zn^2+^ ions interacted with His54, 56, 59, 131, 191 and Asp58 of Bleg1_2478 active site, ampicillin interacted with other residues in the active site namely Leu89 (on α4), Arg159 (on loop 13 which acted as floor, shown as an orange stick) and Arg163 (on loop 15) (Figure A1). The hydrophobic environment surrounding the active site of Bleg1_2478 and ampicillin (Figure A2) helps to retain the substrate in the active site by interacting with the hydrophobic β side chain of ampicillin, similar to observations related to B3 MBLs interactions with β-lactam antibiotics [21,22,34]. The hydrophobic residues were Pro9 and Ile10 (on loop 2, postulated to be part of a doorkeeper structure), Phe57 (on loop 5, the ceiling structure), Phe153 and Ser156 (on loop 13, the floor structure), similar to previous observations made by Tan et al., 2017 [22]. Other than this, π–π, cation–π and hydrophobic interactions formed by His131, Arg159 and Leu89 with the benzene and β-lactam rings of ampicillin further facilitate the binding of the molecule in Bleg1_2478 active site. Other than the metal-binding ligands, none of the other residues mentioned above are well conserved in B3 MBLs.

Non-covalent interactions such as hydrogen bonds, π–π aromatic stacking, cation–π interactions, hydrophobic interactions, halogen bonds, and salt bridges are vital in drug design, particularly to improve the molecular recognition and binding affinity between the protein–ligand interfaces [35]. Taking this into account as well as the hydrophobic nature of the Bleg1_2478 active site, several peptides with hydrophobic residues were screened, designed and further derivatized in silico. In vitro assay showed that two peptides, namely RSWPWH and SSWWDR, inhibited Bleg1_2478 by approximately 50% (IC_50_) at only 0.90 and 0.50 μM, respectively (Figure 4 and Figure 5, Table 5). Almost complete inhibition of Bleg1_2478 was achieved when 10 µM RSWPWH and 20 µM SSWWDR were used. The IC_50_ concentrations of the peptides are lower compared to other inhibitors such as pyrrozole derivative compound [36] and hydroxamic acid derivatives: 2,5-substituted benzophenone hydroxamic acid [37] and cysteine-containing peptides [23]; and are comparable to other reported inhibitors against B3 MBL such as penicillin (β-lactam) derived inhibitors [38], dicarboxylic acid derivatives, i.e., N-heterocyclic dicarboxylic acid [39], and thioester-based inhibitors, i.e., amino acid thioester derivatives [40]. To date, homo-cysteinyl peptide inhibitor recorded the lowest inhibitory concentration of 2 nM [24].

Prediction of possible binding sites of the inhibitory peptides on Bleg1_2478 via random docking showed that RSWPWH might bind to a site slightly away from Bleg1_2478 active site (Figure 7A,B) while SSWWDR might bind to a site that was far away from the active site (Figure 9A,B). It may be unlikely that such binding sites could cause inhibition of Bleg1_2478 due to their distance away from key structural and functional residues important for Bleg1_2478. Fixed docking analyses showed that RSWPWH bound at the vicinity of Bleg1_2478 active site (Figure 8A,B) while SSWWDR bound at the active site itself (Figure 10A,B). Such positionings of the peptides would reduce or block the accessibility of the active site from ampicillin. Analyses of the interactions between the inhibitory peptides and Bleg1_2478 from fixed docking simulations revealed that both peptides bound to several key residues postulated to be important for substrate binding and catalytic activity of Bleg1_2478. They were Phe153, Ser156, His167, His191 and Phe206. Phe153 is a hydrophobic residue in Bleg1_2478 substrate binding site. Hydrophobic residues in the binding cavity of MBLs were predicted to enable the interaction between the enzymes and β-lactams to allow the hydrophobic β side chain of β-lactams into the binding pocket [22,34]. Ser156, on the other hand, was predicted to provide a second shell effect in lodging β-lactam substrates in the binding pocket by forming an extended network of hydrogen bonds with the backbone of nitrogen of Asp58 and His191 of Bleg1_2478 [22]. Hydrogen bonds and hydrophobic interactions can support each other mutually. When a hydrogen bond is present next to the side chain of the ligand, it elevates the strength of the hydrophobic interaction by holding the side chain closer and firmly against the hydrophobic pocket. The improved strength and stabilized geometry of the hydrophobic side chain help to increase the strength of the hydrogen bond [41]. His-191 is a putative Zn^2+^-binding ligand important for catalysis [22]. As these functionally important residues in the active site of Bleg1_2478 are predicted to form interactions with the inhibitory peptides, this, in turn, hindered the binding and hydrolysis of ampicillin by the enzyme, as observed from the results of the inhibition assay. Based on these results, it may be more probable that the inhibitory peptides bind near or at the active site of Bleg1_2478 compared to the secondary sites observed from random docking.

The basic or cationic nature of RSWPWH inhibitory peptide, as well as its predicted binding site at an accessible area of Bleg1_2478 (Figure 8A,B), may have contributed to its ease of interaction with the enzyme, hence, giving forth more favourable and spontaneous binding (Figure 6). However, it may be more exposed to pH changes caused by the cellular environment, making it more susceptible to dissociate from the protein (Table 8). As for SSWWDR, its neutral net charge and its predicted binding site at a less accessible narrow groove of Bleg1_2478 active site (Figure 10A,B) may have resulted in less spontaneous binding (Figure 6). As the binding site is less exposed to the cellular environment, particularly to pH changes, SSWWDR is less susceptible to dissociate from the protein; hence giving forth a K_d_ value that is significantly higher than RSWPWH (Table 6).

Future studies including in vitro stability of the inhibitory peptides, mutational analyses of Bleg1_2478 secondary binding sites and X-ray crystallography of the Bleg1_2478-peptide complexes will be undertaken to gain more detailed insights of the peptides, their binding sites and key interactions involved. This in turn will enable the mechanism of action of the inhibitory peptides to be deduced.

## 4. Materials and Methods

### 4.1. Bacterial Strains and Plasmids

*E. coli* BL21 Star^TM^ (DE3) (Invitrogen, Carlsbad, CA, USA), transformed with previously constructed pET28b(+):Bleg1_2478 recombinant plasmid harbouring bleg1_2478 open reading frame (ORF) (Tan Soo Huei, 2018, unpublished data), was used as the expression host in this study.

### 4.2. Fixed and Random Docking of Ampicillin against Bleg1_2478

**Figure A3:**
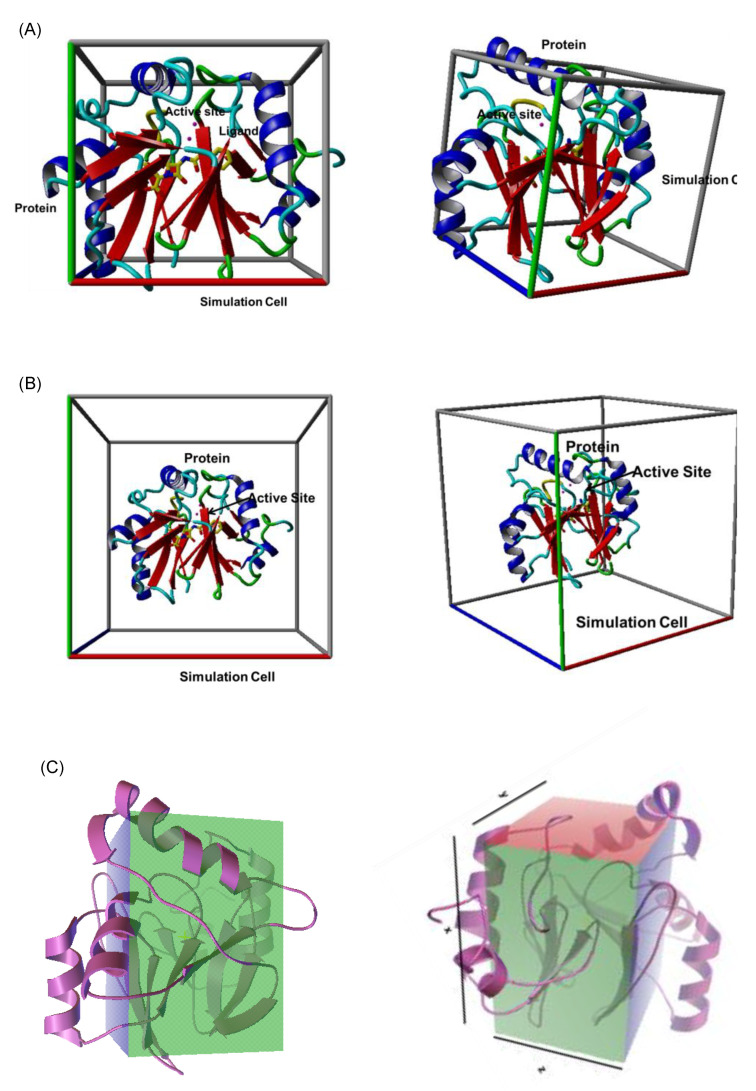
Simulation cell of (**A**) fixed and (**B**) random docking by YASARA; and (**C**) fixed docking using AutoDock Vina.

Previously built dizinc Bleg1_2478 protein model [22] was used as the target protein to design inhibitory peptides. For this purpose, the protein was firstly docked with its preferred substrate, ampicillin, to serve as the benchmark (positive control) to be used for comparison with specially designed inhibitory peptides. The structure of ampicillin was retrieved from Drug Bank database. Docking was performed via fixed and random docking respectively. For fixed docking, YASARA [25] and AutoDock Vina [27] were used. Firstly, the grid box with specific dimensions (for YASARA: x = 32.95, y = 30.99, z = 29.64 Å; α = β = γ = 90°; for AutoDock Vina: x = 80, y = 66, z = 54 Å; center-x = −23.238, center-y = −10.565, center-z = −44.918) was placed surrounding Bleg1_2478 active site which consists of the following putative metal-binding ligands: His-54, His-56, Asp-58, His-59, His-131 and His-191 and other amino acids such as Gln-11, Thr-12, Asn-13, Tyr-15, Asp-28, Pro-129, Gly-130, Asp-150, Phe-153, Ser-156, Ile-157, and Gly-158 (Figure A3A,B); which were predicted to play structural and functional role in Bleg1_2478 [22]. For random docking, YASARA [25] was used. The grid box (x = 55.66, y = 55.46, z = 57.72 Å; α = β = γ = 90°) was set larger to cover all the atoms of the targeted protein (Figure A3C). This was done to identify possible secondary sites on Bleg1_2478, other than the areas surrounding the active site, that the peptides might bind to. Docking results between peptides and Bleg1_2478 were visualized using Pymol V1.7.4 (Schrödinger, New York, NY, USA).

### 4.3. Design and Docking of Inhibitory Peptides against Bleg1_2478

Short antibacterial peptides (AMP) were retrieved from the Collection of Anti-Microbial Peptides (CAMPR3) online database [26] and docked against Bleg1_2478 to determine the binding energy. This was performed by using YASARA [25] and AutoDock Vina [27]. Using those AMPs with acceptable binding energy as templates, inhibitory peptides against Bleg1_2478 were duly designed by altering the amino acid sequences of the AMPs and their binding efficiency (energy) to Bleg1_2478 was analysed via fixed and random docking respectively using YASARA [25]. The binding energy of ampicillin (the preferred substrate) is set as the benchmark to select designed peptides that portray equivalent or better binding energies. The design and docking process was repeated a few times to obtain a pool of derivatised peptides with favourable binding energy to serve as potential inhibitors of Bleg1_2478. The selected derivatised peptides were docked again using AutoDock Vina [27] to validate the result. All the designed peptides with desirable binding energy were synthesised by Mimotopes Pty Ltd. with more than 95% purity.

### 4.4. Overexpression and Purification of Bleg1_2478 Recombinant Protein

Heterologous production and purification of Bleg1_2478 recombinant protein were performed following the method by [22] with slight modifications. Luria–Bertani (LB) broth (10 mL) containing 50 µg/mL of kanamycin was inoculated with recombinant *E. coli* BL21 Star^TM^ (DE3)::Bleg1_2478 and cultivated for 18 h at 37 °C. Following this, 2 mL of the starter culture was transferred to a fresh 200 mL of LB medium containing 50 µg/mL of kanamycin and 100 µM of ZnSO_4_. This culture was cultivated until its optical density at 600 nm (OD_600_) reached 0.6–0.8. At this time, isopropyl-β-d-thiogalactopyranoside (IPTG) was added into the culture to a final concentration of 0.1 mM to induce the production of the recombinant protein. After induction, the culture was further cultivated at 20 °C for 20 h. Cells were then harvested by centrifugation at 9000× *g* for 20 min at 4 °C. The cell pellet was resuspended in 5 mL of binding buffer (Buffer A: 20 mM phosphate buffer, 0.5 M NaCl, 50 mM imidazole, 2 mM MgSO_4_) (pH 7.4). Cells were then disrupted via sonication with 30% amplitude, 15 s ON and 15 s OFF pulses for 2 min 30 s on ice. The lysate was centrifuged at 9000× *g* for 20 min at 4 °C. The soluble and insoluble fractions were collected and analysed with 12% (*w*/*v*) sodium dodecyl sulphate polyacrylamide electrophoresis (SDS PAGE). Soluble fraction containing the N-terminal His-tagged protein was filtered through a 0.45 µM hydrophilic membrane before subjected to purification via affinity chromatography. The filtrate was loaded into charged nickel-nitriloacetic acid (Ni^2+^-NTA) column, pre-equilibrated with 5 column volumes (CV) of Buffer A at 1 mL/min, before sample injection. Recombinant Bleg1_2478 protein was then eluted with elution buffer (Buffer B: 20 mM sodium phosphate buffer (Na_2_HPO_4_-NaH_2_PO_4_), 0.5 M sodium chloride (NaCl) and 0.5 M imidazole) (pH 7.4). Subsequently, the protein was dialysed against storage buffer (Buffer C: 20 mM Na_2_HPO_4_-NaH_2_PO_4_, 100 µM ZnSO_4_, and 5% (*v/v*) glycerol) (pH 7.4) at protein to buffer ratio of 1:100. Snakeskin dialysis tube (Thermofisher) with a cutoff size of 10 kDa was used for the process. Protein samples were analysed with SDS-PAGE and subjected to Bradford assay [42] using bovine serum albumin (BSA) as the standard.

### 4.5. Inhibitory Assay of Designed Peptides against Bleg1_2478

The ability of the peptides in inhibiting Bleg1_2478 was investigated via inhibitory assay. Firstly, enzymatic assay of purified Bleg1_2478 recombinant protein using ampicillin as the substrate was performed, serving as the positive control. The assay was carried out based on the method by [18]. All assays were performed at 30 °C in total assay volume of 3 mL containing 20 mM sodium phosphate (Na_2_HPO_4_-NaH_2_PO_4_) (pH 7.0), 20 µg/mL BSA, 100 µM ZnSO_4_, 10 µM Bleg1_2478 and 100 µM ampicillin. The reaction was monitored by recording the absorbance at 235 nm every 1 min for a total duration of 6 min using a 50 Bio UV Visible Spectrophotometry. For inhibitory assay of the designed peptides, the assay above was performed with the addition of the peptides into the assay mixture at concentrations of 1, 10 and 20 µM. All assays were done in triplicates. Following this, peptides that showed significant inhibition on Bleg1_2478 were further assayed at a concentration below 1 µM to determine the least concentration needed to exert the slightest inhibition of Bleg1_2478.

### 4.6. Isothermal Titration Calorimetry (ITC) Analysis of Inhibitory Peptides with Bleg1_2478

ITC measurement was performed at 30 °C with nanoisothermal titration calorimetry, TA Instrument, USA at Malaysian Genome Institute. The peptides were dissolved in 20 mM sodium phosphate buffer (Na_2_HPO_4_-NaH_2_PO_4_) (pH 7.0). Bleg1_2478 protein sample, the selected peptide inhibitors and 20 mM sodium phosphate buffer (Na_2_HPO_4_-NaH_2_PO_4_) (pH 7.0) were firstly degassed by using Themovac Unit. The experiment was carried out at 30 °C with stirring speed of 300 rpm. The calorimetry data were subsequently analysed using NanoAnalyze Software v3.8.0. (New Castle, DE, USA)

### 4.7. Physicochemical Predictions of Peptides

Online tools were used to predict the physicochemical properties of the peptides. Genescript tool (https://www.genscript.com/tools/peptide-property-calculator) [28] was used to predict net charge; APD3: Calctool (http://www.calctool.org/CALC/prof/bio/protein_length) [29] was used to determine the diameter of the peptides; Peptide Property Calculator [30] to predict the approximate volume; Antimicrobial Peptide Calculator and Predictor [31] was used to determine total hydrophobic ratio; Bachem–Peptide Calculator [32] was used to calculate average hydrophilicity and ExPaSy ProtParam [33] was used to determine instability index of the peptides.

## Figures and Tables

**Table 1:** Interacting residues of Bleg1_2478 with ampicillin, the types of interactions and distance between the residues obtained from fixed docking.

Bleg1_2478	Ampicillin	Types of Interactions	Distance (Å)
CB Leu89	C6	Hydrophobic	3.7
NE2 His131	H11	π–π	3.0
HH1 Arg159	C15	Cation–π	2.9
Zn1	C11	Cation–π	3.5

**Table 2:** Interacting residues of Bleg1_2478 with ampicillin, the types of interactions and distance between the residues obtained from random docking.

Bleg1_2478	Ampicillin	Types of Interactions	Distance (Å)
CD Arg159	C13	Hydrophobic	3.5
HH1 Arg159	C15	Cation–π	2.6
NE2 His131	H11	π–π	3.1
HE Arg163	O2	H-bond	2.6
Zn1	C13	Cation–π	4.1

**Table 3:** Binding energies of peptides (retrieved from CAMPR3 database) generated when randomly docked to Bleg1_2478 using YASARA.

Peptide	Binding Energy (kcal/mol)
RRYYRF	6.94
KKWWKF	6.57
RRAARF	8.83
MRTGNAN	5.71
WLAFVLV	5.22
NRPVYIPRPP	2.95
RRWFWR	7.50
RRWWFR	7.88

**Table 4:** The docking of derived peptides with Bleg1_2478 via YASARA and AutoDock Vina.

Peptide Sequence	Binding Energy (kcal/mol) YASARA		Binding Affinity (kcal/mol) AutoDock Vina Fixed Docking
	Fixed Docking	Random Docking	
Ampicillin (control)	8.52	8.15	−7.0
RYWPRF	9.86	7.42	−8.0
RYTPRF	7.89	7.02	−7.4
RSWNWH	7.26	8.89	−8.4
RSWCWH	7.93	9.01	−7.2
RSWPWH	8.93	8.34	−8.2
RRWWSR	No binding	7.83	−8.4
SRWWDR	6.15	6.90	−8.1
SRWWYR	6.96	8.21	−8.2
SSWWDR	6.47	6.15	−7.5

**Table 5:** IC_50_ values of RSWPWH and SSWWDR against Bleg1_2478.

Peptide	IC_50_ (µM) ^(a)^
RSWPWH	0.90 ± 0.17
SSWWDR	0.50 ± 0.02

^(a)^ IC_50_ was determined from enzymatic assays performed at 30 °C in 3 mL reaction mixture containing 20 mM sodium phosphate (Na_2_HPO_4_-NaH_2_PO_4_) (pH 7.0), 10 µM Bleg1_2478, 100 µM ampicillin, 100 µM ZnSO_4_ and 20 µg/mL BSA. Assays were performed in triplicates.

**Table 6:** Thermodynamic parameters determined for the interaction of ampicillin and inhibitory peptides with Bleg1_2478.

Ligands	Association Constant, K_a_ (M^−1^)	Dissociation Constant, K_d_ (M)	Stoichiometry, n	Entropy, ΔS (J/mol·K)
Ampicillin (positive control)	0.68 × 10^6^	1.47 × 10^−6^	0.928	−248.00
RSWPWH	2.01 × 10^6^	4.97 × 10^−7^	0.941	−54.35
SSWWDR	1.01 × 10^6^	9.92 × 10^−7^	0.722	−29.10

**Table 7:** The types of interactions between the residues of protein and peptide RSWPWH and their distances respectively.

Types of Docking	Atoms and Residues Involved in the Interaction		Types of Interaction	Distance(Å)
	Protein	RSWPWH		
Random docking	CB Ser92	CH2 Trp3	Hydrophobic	3.6
	CE3 Trp83	NE1 Trp5	π–π	3.5
	NE Arg163	CD1 Trp3	Cation–π	4.1
	OE2 Glu85	HE Arg1	H-Bond	1.7
	OE1 Glu85	HH2 Arg1	H-Bond	1.9
Fixed docking	HB Phe153	CH2 Trp3	Hydrophobic	2.6
	ND1 His167	CZ2 Trp5	π–π	4.0
	CE2 Phe206	NH2 Arg1	Cation–π	3.3
	HG Ser156	O Trp3	H-Bond	2.0

**Table 8:** The types of interactions between the residues of protein and peptide SSWWDR and their distances respectively.

Types of Docking	Atoms and Residues Involved in the Interaction		Types of Interaction	Distance(Å)
	Protein	SSWWDR		
Random docking	CD 2 Tyr18	CZ2 Trp3	Hydrophobic	3.4
	CE 2 Tyr18	CZ2 Trp3	π–π	2.9
	O Tyr18	NE1 Trp3	H–Bond	2.2
	H Met1	O Ser2	H–Bond	2.1
	HH Tyr140	O Trp3	H–Bond	1.8
	O Lys142	HE1 Arg6	H–Bond	2.5
Fixed docking	CE2 Phe153	CE2 Trp4	Hydrophobic	3.6
	CD2 His191	CD1 Trp4	π–π	3.2

**Table 9:** Predicted physicochemical properties of RSWPWH and SSWWDR inhibitory peptides.

Peptide	Net Charge ^(a)^	Molecular Weight ^(a)^	Diameter (nm) ^(b)^	Approximate Volume (A^3^) ^(c)^	Total Hydrophobic Ratio (%) ^(d)^	Average Hydrophilicity ^(e)^	Instability Index ^(f)^
RSWPWH	+2, basic	867.96	1.33	1050	33	−0.7	116.23
SSWWDR	0, neutral	835.87	1.31	1012	33	0.0	27.87

(^a^) Predicted using Genescript’s Peptide Molecular Weight Calculator [28]. (^b^) Predicted using Calctool [29]. (^c^) Predicted using Peptide Property Calculator [30]. (^d^) Predicted using APD3: Antimicrobial Peptide Calculator and Predictor [31]. (^e^) Predicted using Bachem–Peptide Calculator [32]. (^f^) Predicted using ExPaSy ProtParam [33].

## Abbreviations

MBL	Metallo-β-lactamase
SBL	Serine-β-lactamase
AMR	Antimicrobial resistance
ITC	Isothermal titration calorimetry
